# 1-(3-Fluoro­phen­yl)-4,4,6-trimethyl-3,4-dihydro­pyrimidine-2(1*H*)-thione

**DOI:** 10.1107/S1600536811023671

**Published:** 2011-06-25

**Authors:** Bohari M. Yamin, Ruhana L. Lawi, Halima F. Salem

**Affiliations:** a School of Chemical Sciences and Food Technology, Univeriti Kebangsaan Malaysia, UKM 43600 Bangi Selangor, Malaysia

## Abstract

In the title compound, C_13_H_15_FN_2_S, the dihydro­pyrimidine ring is essentially planar, with a maximum deviation of 0.086 (3) Å from the mean plane of the rest of the ring for the dimethyl­ated C atom. The benzene ring is almost perpendicular to the dihydro­pyrimidine ring, with a dihedral angle of 83.97 (14)°. The crystal packing is characterized by centrosymmetric dimers resulting from pairs of inter­molecular N—H⋯S hydrogen bonds. There are also C—H⋯π inter­actions.

## Related literature

For the biological properties of related compounds, see: Rovnyak *et al.* (1995 ▶); Kappe (2000 ▶); Alam *et al.* (2005 ▶); Sriram *et al.* (2006 ▶); Leite *et al.* (2006 ▶). For related structures, see: Yamin *et al.* (2005 ▶); Ismail *et al.* (2007 ▶); Saeed *et al.* (2010 ▶); Yamin & Salem (2011 ▶). For standard bond lengths, see: Allen *et al.* (1987 ▶).
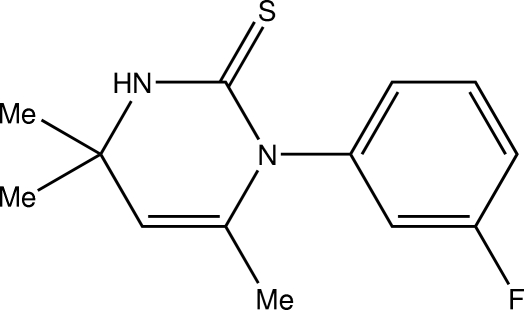


## Experimental

### 

#### Crystal data

C_13_H_15_FN_2_S
*M* *_r_* = 250.33
Monoclinic, 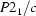

*a* = 8.814 (3) Å
*b* = 14.997 (5) Å
*c* = 10.215 (3) Å
β = 95.711 (6)°
*V* = 1343.6 (7) Å^3^
*Z* = 4
Mo *K*α radiation
μ = 0.23 mm^−1^
*T* = 298 K
0.50 × 0.29 × 0.20 mm

#### Data collection

Bruker SMART APEX CCD area-detector diffractometer
Absorption correction: multi-scan (*SADABS*; Bruker, 2000 ▶) *T*_min_ = 0.892, *T*_max_ = 0.954
7116 measured reflections
2497 independent reflections
1764 reflections with *I* > 2σ(*I*)
*R*_int_ = 0.034

#### Refinement

*R*[*F* ^2^ > 2σ(*F* ^2^)] = 0.059
*wR*(*F* ^2^) = 0.153
*S* = 1.06
2497 reflections
157 parameters
H-atom parameters constrained
Δρ_max_ = 0.37 e Å^−3^
Δρ_min_ = −0.17 e Å^−3^

### 

Data collection: *SMART* (Bruker, 2000 ▶); cell refinement: *SAINT* (Bruker, 2000 ▶); data reduction: *SAINT*; program(s) used to solve structure: *SHELXS97* (Sheldrick, 2008 ▶); program(s) used to refine structure: *SHELXL97* (Sheldrick, 2008 ▶); molecular graphics: *SHELXTL* (Sheldrick, 2008 ▶); software used to prepare material for publication: *SHELXTL*, *PARST* (Nardelli, 1995 ▶) and *PLATON* (Spek, 2009 ▶).

## Supplementary Material

Crystal structure: contains datablock(s) global, I. DOI: 10.1107/S1600536811023671/ld2016sup1.cif

Structure factors: contains datablock(s) I. DOI: 10.1107/S1600536811023671/ld2016Isup2.hkl

Supplementary material file. DOI: 10.1107/S1600536811023671/ld2016Isup3.cml

Additional supplementary materials:  crystallographic information; 3D view; checkCIF report

## Figures and Tables

**Table 1:** Hydrogen-bond geometry (Å, °) *Cg*1 is the centroid of the N1/N2/C1–C4 pyrimidine ring.

*D*—H⋯*A*	*D*—H	H⋯*A*	*D*⋯*A*	*D*—H⋯*A*
N1—H1*A*⋯S1^i^	0.86	2.57	3.400 (3)	162
C9—H9*A*⋯*Cg*1^ii^	0.93	2.89	3.788 (4)	163

Symmetry codes: (i) 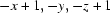; (ii) 
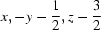
.

**Fig. 1.**
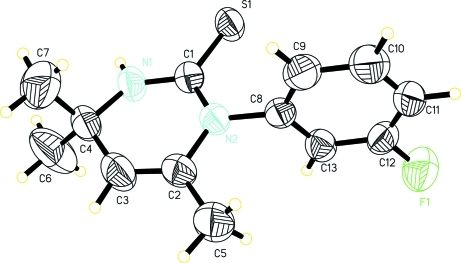
Molecular structure of (1), with the atomic-labelling scheme. Displacement ellipsoid are drawn at the 50% probablity level.

**Fig. 2.**
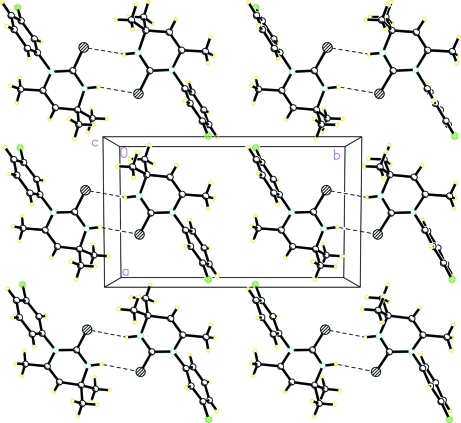
The packing of (1) viewed down the c axis. Hydrogen bonds are shown by dashed lines.

## supplementary crystallographic information

### Comment

Pyrimidine-2(1*H*)-ones/thiones are calcium channel blocker compounds (Rovnyak *et al.*, 1995). They also have other biological activities such as antibacterial, antifungal and antiviral (Kappe, 2000; Alam *et al.*, 2005; Sriram *et al.*, 2006; Leite *et al.*, 2006). The 4,4,6-trimethyl-1-aryl-3,4-dihydropyrimidine-2-(1*H*)-thiones open a new series of 3,4-dihydro pyrimidine-2-(1*H*)-thione derivatives following publication of 4,4,6- trimethyl-1-phenyl-3,4-dihydropyrimidine-2-(1*H*)-thione (Yamin *et al.*, 2005; Ismail *et al.*, 2007). The title compound is isomorphous to 4,4,6-trimethyl-1-(3-chlorophenyl)-3,4-dihydropyrimidine-2-(1*H*)-thione (Yamin & Salem, 2011) and 4,4,6-trimethyl-1-(3-methylphenyl)-3,4-dihydropyrimidine-2-(1*H*)-thione (Saeed *et al.*, 2010). The dihydropyrimidine (N1,N2,C1—C4) ring is planar with maximum deviation of 0.086 (3)Å for C4 atom from the least square plane. The benzene ring is perpendicular to the dihydropyrimidine with dihedral angle of 83.97 (14)°, slightly smaller than that in the *meta*- chloro analog (86.62 (13)°). The bond lenghts and angles are in normal ranges (Allen *et al.*, 1987) and are comparable to those in the above mentioned analogs. In the crystal, the molecules are linked by N1—H1A···S1 intermolecular hydrogen bonds (see symmetry code in Table 2) to form centrosymmetric dimers parallel to the *ab* face (Fig 2). There is also a C9—H9A···π interaction involving the pyrimidine (C_g_1: N1/N2/(C1—C4)) ring (Table 2).

### Experimental

A procedure similar to that used for the preparation of 4,4,6-Trimethyl-1-(3-chlorophenyl)-3,4-dihydropyrimidine-2-(1*H*)-thione (Yamin & Salem,2011) was followed. Equimolar quantities of thiocyanic acid and 3-fluoroaniline (5.4 mmol) in acetone were stirred for 2–3 h. Colourless crystals of 78% yield were obtained after 3 days by evaporation at room temperature. Melting point 456.8–458.9 K.

### Refinement

H atoms on the C and N atoms were positioned geometrically with C—H= 0.93 (aromatic and olefinic), 0.96 Å (methyl) and N—H = 0.86 Å respectively, and constrained to ride and rotate (for Me groups) on their parent atoms with *U*_iso_=*x*_eq_(parent atom) where *x*=1.2 for N, aromatic C and olefinic C and *x*=1.5 for methyl C. There is a highest peak and deepest hole of 0.45 from H10 and 0.76Å from F1 atom the respectively.

### Crystal data

**Table:**

C_13_H_15_FN_2_S	*F*(000) = 528
*M**_r_* = 250.33	*D*_x_ = 1.238 Mg m^−^^3^
Monoclinic, *P*2_1_/*c*	Melting point = 458.9–456.8 K
Hall symbol: -P 2ybc	Mo *K*α radiation, λ = 0.71073 Å
*a* = 8.814 (3) Å	Cell parameters from 2497 reflections
*b* = 14.997 (5) Å	θ = 2.3–25.5°
*c* = 10.215 (3) Å	µ = 0.23 mm^−^^1^
β = 95.711 (6)°	*T* = 298 K
*V* = 1343.6 (7) Å^3^	Block, colourless
*Z* = 4	0.50 × 0.29 × 0.20 mm

### Data collection

**Table:**

Bruker SMART APEX CCD area-detector diffractometer	2497 independent reflections
Radiation source: fine-focus sealed tube	1764 reflections with *I* > 2σ(*I*)
graphite	*R*_int_ = 0.034
ω scans	θ_max_ = 25.5°, θ_min_ = 2.3°
Absorption correction: multi-scan (*SADABS*; Bruker, 2000)	*h* = −10→10
*T*_min_ = 0.892, *T*_max_ = 0.954	*k* = −16→18
7116 measured reflections	*l* = −12→11

### Refinement

**Table:**

Refinement on *F*^2^	Primary atom site location: structure-invariant direct methods
Least-squares matrix: full	Secondary atom site location: difference Fourier map
*R*[*F*^2^ > 2σ(*F*^2^)] = 0.059	Hydrogen site location: inferred from neighbouring sites
*wR*(*F*^2^) = 0.153	H-atom parameters constrained
*S* = 1.06	*w* = 1/[σ^2^(*F*_o_^2^) + (0.0666*P*)^2^ + 0.4935*P*] where *P* = (*F*_o_^2^ + 2*F*_c_^2^)/3
2497 reflections	(Δ/σ)_max_ = 0.001
157 parameters	Δρ_max_ = 0.37 e Å^−^^3^
0 restraints	Δρ_min_ = −0.17 e Å^−^^3^

### Special details

**Table:**

Geometry. All e.s.d.'s (except the e.s.d. in the dihedral angle between two l.s. planes) are estimated using the full covariance matrix. The cell e.s.d.'s are taken into account individually in the estimation of e.s.d.'s in distances, angles and torsion angles; correlations between e.s.d.'s in cell parameters are only used when they are defined by crystal symmetry. An approximate (isotropic) treatment of cell e.s.d.'s is used for estimating e.s.d.'s involving l.s. planes.
Refinement. Refinement of *F*^2^ against ALL reflections. The weighted *R*-factor *wR* and goodness of fit *S* are based on *F*^2^, conventional *R*-factors *R* are based on *F*, with *F* set to zero for negative *F*^2^. The threshold expression of *F*^2^ > σ(*F*^2^) is used only for calculating *R*-factors(gt) *etc*. and is not relevant to the choice of reflections for refinement. *R*-factors based on *F*^2^ are statistically about twice as large as those based on *F*, and *R*- factors based on ALL data will be even larger.

### Fractional atomic coordinates and isotropic or equivalent isotropic displacement parameters (Å^2^)

**Table:**

	*x*	*y*	*z*	*U*_iso_*/*U*_eq_	
F1	0.9699 (2)	0.40143 (14)	0.6648 (2)	0.0938 (7)	
S1	0.65807 (8)	0.11011 (5)	0.47356 (8)	0.0623 (3)	
N1	0.3831 (3)	0.11471 (16)	0.5539 (2)	0.0620 (7)	
H1A	0.3939	0.0577	0.5561	0.074*	
N2	0.4991 (2)	0.25140 (15)	0.5427 (2)	0.0519 (6)	
C1	0.5038 (3)	0.1608 (2)	0.5251 (3)	0.0506 (7)	
C2	0.3725 (3)	0.2932 (2)	0.5938 (3)	0.0601 (8)	
C3	0.2523 (4)	0.2442 (2)	0.6138 (4)	0.0780 (10)	
H3A	0.1727	0.2723	0.6507	0.094*	
C4	0.2343 (3)	0.1480 (2)	0.5824 (3)	0.0644 (8)	
C5	0.3852 (4)	0.3902 (2)	0.6208 (4)	0.0895 (12)	
H5A	0.2904	0.4120	0.6471	0.134*	
H5B	0.4085	0.4209	0.5428	0.134*	
H5C	0.4650	0.4006	0.6902	0.134*	
C6	0.1856 (5)	0.0964 (3)	0.6999 (5)	0.1206 (18)	
H6A	0.2611	0.1035	0.7736	0.181*	
H6B	0.1757	0.0343	0.6777	0.181*	
H6C	0.0895	0.1188	0.7222	0.181*	
C7	0.1209 (5)	0.1335 (3)	0.4636 (5)	0.1154 (16)	
H7A	0.1512	0.1673	0.3908	0.173*	
H7B	0.0217	0.1525	0.4832	0.173*	
H7C	0.1177	0.0713	0.4412	0.173*	
C8	0.6272 (3)	0.30394 (18)	0.5099 (3)	0.0493 (7)	
C9	0.6358 (4)	0.3306 (2)	0.3822 (3)	0.0653 (8)	
H9A	0.5581	0.3160	0.3174	0.078*	
C10	0.7597 (5)	0.3789 (2)	0.3506 (4)	0.0802 (11)	
H10A	0.7663	0.3960	0.2638	0.096*	
C11	0.8726 (4)	0.4018 (2)	0.4449 (4)	0.0747 (10)	
H11A	0.9569	0.4341	0.4238	0.090*	
C12	0.8593 (3)	0.3763 (2)	0.5706 (3)	0.0606 (8)	
C13	0.7392 (3)	0.32727 (19)	0.6068 (3)	0.0538 (7)	
H13A	0.7338	0.3104	0.6938	0.065*	

### Atomic displacement parameters (Å^2^)

**Table:**

	*U*^11^	*U*^22^	*U*^33^	*U*^12^	*U*^13^	*U*^23^
F1	0.0710 (13)	0.0981 (17)	0.1111 (16)	−0.0189 (11)	0.0028 (12)	−0.0109 (12)
S1	0.0536 (4)	0.0493 (5)	0.0878 (6)	−0.0031 (3)	0.0263 (4)	−0.0071 (4)
N1	0.0512 (14)	0.0491 (15)	0.0893 (18)	−0.0086 (11)	0.0249 (13)	−0.0059 (13)
N2	0.0487 (12)	0.0461 (14)	0.0630 (14)	−0.0021 (11)	0.0151 (11)	−0.0044 (11)
C1	0.0491 (15)	0.0509 (18)	0.0527 (16)	−0.0045 (13)	0.0093 (13)	−0.0007 (13)
C2	0.0531 (17)	0.0572 (19)	0.0717 (19)	0.0065 (14)	0.0151 (15)	−0.0071 (16)
C3	0.0562 (18)	0.072 (2)	0.110 (3)	0.0044 (17)	0.0322 (19)	−0.011 (2)
C4	0.0487 (16)	0.066 (2)	0.082 (2)	−0.0057 (15)	0.0223 (16)	−0.0062 (17)
C5	0.073 (2)	0.063 (2)	0.137 (3)	0.0071 (17)	0.032 (2)	−0.021 (2)
C6	0.125 (4)	0.109 (4)	0.142 (4)	0.007 (3)	0.086 (3)	0.021 (3)
C7	0.071 (3)	0.134 (4)	0.137 (4)	−0.004 (2)	−0.010 (3)	−0.038 (3)
C8	0.0523 (16)	0.0392 (16)	0.0582 (17)	−0.0004 (12)	0.0142 (14)	−0.0044 (13)
C9	0.079 (2)	0.060 (2)	0.0577 (18)	−0.0084 (17)	0.0093 (16)	−0.0043 (15)
C10	0.116 (3)	0.059 (2)	0.071 (2)	−0.019 (2)	0.034 (2)	0.0013 (17)
C11	0.090 (2)	0.053 (2)	0.088 (3)	−0.0223 (17)	0.042 (2)	−0.0133 (18)
C12	0.0552 (17)	0.0483 (18)	0.079 (2)	−0.0063 (14)	0.0102 (16)	−0.0146 (15)
C13	0.0556 (16)	0.0470 (17)	0.0605 (17)	−0.0005 (13)	0.0138 (14)	0.0029 (14)

### Geometric parameters (Å, °)

**Table:**

F1—C12	1.353 (3)	C6—H6A	0.9600
S1—C1	1.687 (3)	C6—H6B	0.9600
N1—C1	1.326 (3)	C6—H6C	0.9600
N1—C4	1.460 (4)	C7—H7A	0.9600
N1—H1A	0.8600	C7—H7B	0.9600
N2—C1	1.372 (4)	C7—H7C	0.9600
N2—C2	1.423 (3)	C8—C13	1.372 (4)
N2—C8	1.443 (3)	C8—C9	1.374 (4)
C2—C3	1.321 (4)	C9—C10	1.376 (5)
C2—C5	1.483 (4)	C9—H9A	0.9300
C3—C4	1.483 (5)	C10—C11	1.359 (5)
C3—H3A	0.9300	C10—H10A	0.9300
C4—C7	1.509 (5)	C11—C12	1.356 (5)
C4—C6	1.526 (5)	C11—H11A	0.9300
C5—H5A	0.9600	C12—C13	1.369 (4)
C5—H5B	0.9600	C13—H13A	0.9300
C5—H5C	0.9600		
			
C1—N1—C4	128.5 (3)	H6A—C6—H6B	109.5
C1—N1—H1A	115.7	C4—C6—H6C	109.5
C4—N1—H1A	115.7	H6A—C6—H6C	109.5
C1—N2—C2	121.3 (2)	H6B—C6—H6C	109.5
C1—N2—C8	118.4 (2)	C4—C7—H7A	109.5
C2—N2—C8	120.3 (2)	C4—C7—H7B	109.5
N1—C1—N2	116.8 (2)	H7A—C7—H7B	109.5
N1—C1—S1	121.6 (2)	C4—C7—H7C	109.5
N2—C1—S1	121.5 (2)	H7A—C7—H7C	109.5
C3—C2—N2	118.8 (3)	H7B—C7—H7C	109.5
C3—C2—C5	124.3 (3)	C13—C8—C9	120.5 (3)
N2—C2—C5	116.9 (3)	C13—C8—N2	119.7 (2)
C2—C3—C4	125.3 (3)	C9—C8—N2	119.8 (3)
C2—C3—H3A	117.4	C8—C9—C10	119.8 (3)
C4—C3—H3A	117.4	C8—C9—H9A	120.1
N1—C4—C3	107.3 (2)	C10—C9—H9A	120.1
N1—C4—C7	109.1 (3)	C11—C10—C9	120.6 (3)
C3—C4—C7	111.3 (3)	C11—C10—H10A	119.7
N1—C4—C6	108.1 (3)	C9—C10—H10A	119.7
C3—C4—C6	110.9 (3)	C12—C11—C10	118.3 (3)
C7—C4—C6	110.1 (3)	C12—C11—H11A	120.9
C2—C5—H5A	109.5	C10—C11—H11A	120.9
C2—C5—H5B	109.5	F1—C12—C11	118.0 (3)
H5A—C5—H5B	109.5	F1—C12—C13	118.7 (3)
C2—C5—H5C	109.5	C11—C12—C13	123.3 (3)
H5A—C5—H5C	109.5	C12—C13—C8	117.5 (3)
H5B—C5—H5C	109.5	C12—C13—H13A	121.2
C4—C6—H6A	109.5	C8—C13—H13A	121.2
C4—C6—H6B	109.5		
			
C4—N1—C1—N2	10.7 (4)	C2—C3—C4—C7	−107.0 (4)
C4—N1—C1—S1	−171.0 (2)	C2—C3—C4—C6	130.1 (4)
C2—N2—C1—N1	2.1 (4)	C1—N2—C8—C13	−96.2 (3)
C8—N2—C1—N1	−178.8 (2)	C2—N2—C8—C13	82.9 (3)
C2—N2—C1—S1	−176.1 (2)	C1—N2—C8—C9	84.1 (3)
C8—N2—C1—S1	2.9 (3)	C2—N2—C8—C9	−96.8 (3)
C1—N2—C2—C3	−5.8 (4)	C13—C8—C9—C10	1.8 (5)
C8—N2—C2—C3	175.2 (3)	N2—C8—C9—C10	−178.5 (3)
C1—N2—C2—C5	174.3 (3)	C8—C9—C10—C11	−1.1 (5)
C8—N2—C2—C5	−4.7 (4)	C9—C10—C11—C12	−0.4 (5)
N2—C2—C3—C4	−2.6 (5)	C10—C11—C12—F1	−178.1 (3)
C5—C2—C3—C4	177.3 (4)	C10—C11—C12—C13	1.2 (5)
C1—N1—C4—C3	−16.9 (4)	F1—C12—C13—C8	178.9 (2)
C1—N1—C4—C7	103.7 (4)	C11—C12—C13—C8	−0.5 (5)
C1—N1—C4—C6	−136.5 (3)	C9—C8—C13—C12	−1.0 (4)
C2—C3—C4—N1	12.2 (5)	N2—C8—C13—C12	179.3 (2)

### Hydrogen-bond geometry (Å, °)

**Table:**

Cg1 is the centroid of the N1/N2/C1–C4 pyrimidine ring.

**Table:**

*D*—H···*A*	*D*—H	H···*A*	*D*···*A*	*D*—H···*A*
N1—H1A···S1^i^	0.86	2.57	3.400 (3)	162
C9—H9A···Cg1^ii^	0.93	2.89	3.788 (4)	163

Symmetry codes: (i) −*x*+1, −*y*, −*z*+1; (ii) *x*, −*y*−1/2, *z*−3/2.
